# Archaeological and Contemporary Evidence Indicates Low Sea Otter Prevalence on the Pacific Northwest Coast During the Late Holocene

**DOI:** 10.1007/s10021-021-00671-3

**Published:** 2021-08-17

**Authors:** Erin Slade, Iain McKechnie, Anne K. Salomon

**Affiliations:** 1 School of Resource and Environmental Management, Simon Fraser University, Burnaby, British Columbia V5A 1S6 Canada; 2 Historical Ecology & Coastal Archaeology Laboratory, Department of Anthropology, University of Victoria, Cornett B246a, 3800 Finnerty Rd, Victoria, British Columbia V8P 5C2 Canada; 3 Hakai Institute, Heriot Bay, Quadra Island, British Columbia Canada; 4 Bamfield Marine Sciences Centre, Bamfield, British Columbia V0R 1B0 Canada

**Keywords:** historical ecology, archaeology, Indigenous knowledge, shifting baselines, marine ecology, sea otters

## Abstract

The historic extirpation and subsequent recovery of sea otters (*Enhydra lutris*) have profoundly changed coastal social-ecological systems across the northeastern Pacific. Today, the conservation status of sea otters is informed by estimates of population carrying capacity or growth rates independent of human impacts. However, archaeological and ethnographic evidence suggests that for millennia, complex hunting and management protocols by Indigenous communities limited sea otter abundance near human settlements to reduce the negative impacts of this keystone predator on shared shellfish prey. To assess relative sea otter prevalence in the Holocene, we compared the size structure of ancient California mussels (*Mytilus californianus*) from six archaeological sites in two regions on the Pacific Northwest Coast, to modern California mussels at locations with and without sea otters. We also quantified modern mussel size distributions from eight locations on the Central Coast of British Columbia, Canada, varying in sea otter occupation time. Comparisons of mussel size spectra revealed that ancient mussel size distributions are consistently more similar to modern size distributions at locations with a prolonged absence of sea otters. This indicates that late Holocene sea otters were maintained well below carrying capacity near human settlements as a result of human intervention. These findings illuminate the conditions under which sea otters and humans persisted over millennia prior to the Pacific maritime fur trade and raise important questions about contemporary conservation objectives for an iconic marine mammal and the social-ecological system in which it is embedded.

**Supplementary Information:**

The online version contains supplementary material available at 10.1007/s10021-021-00671-3.

## Highlights

- Indigenous peoples mediated the keystone role of sea otters in nearshore ecosystems.
- Large mussels in ancient human settlements indicate sea otters were rare or absent.
- Millennia of interactions with humans constrained the realized niche of sea otters.

## Introduction

In 1998, Paul Dayton, Mia Tegner, and colleagues identified a challenge at the crux of endangered species management: population baselines cannot be defined without considering potential ecological ‘ghosts’ that served formerly consequential roles in marine ecosystems. Resource managers and coastal communities have since been challenged to extend baselines from which to measure ecosystem change, and increasingly acknowledge the importance of archaeological and anthropological data to broaden perspectives and identify changes beyond the time scales of direct ecological observation which typically only span recent decades (Pauly 1995; Dayton and others 1998). Long-term data sets are particularly important in marine systems where keystone predators have been eliminated (McCauley and others 2015) or have had their ecological role greatly diminished before inclusion in modern management and restoration targets (Dayton and others 1998; Jackson and others 2001). There is also a growing acknowledgement of the functional role that humans have played in food webs globally (Salomon and others 2010; Boivin and others 2016; Dunne and others 2016; Worm and Paine 2016), particularly the resource harvesting practices of Indigenous peoples over millennia that have greatly diminished since European contact, contributing to misperceptions of ‘natural’ ecological baselines (Bliege Bird and Nimmo 2018; Power and others 2018; Ellis and others 2021).

In Canada, an emerging challenge exists between the recovery and protection status of sea otters, a well-recognized shellfish predator, and the constitutionally protected rights of Indigenous people to access those same shellfish. The crux of this conservation and management conflict hinges on our perspective of historical baselines and the role humans once played, and continue to play, in coastal ecosystems (Salomon and others 2015; Pinkerton and others 2019; Burt and others 2020). Currently, sea otters (*Enhydra lutris*) in Canada are protected under the federal *Fisheries Act (*R.S.C., 1985*)*, and their recovery is defined as occurring when their 'long-term persistence in the wild is secured' (Sea Otter Recovery Team 2007). Functionally, their conservation status is determined by their population trend over three generations (COSEWIC 2007). In the USA, sea otter conservation targets are scaled to a conceptual population carrying capacity. Both estimates, however, are independent of human impacts and conservation targets are implicitly viewed as 'to allow full, pre-exploitation recovery' (Davis and others 2019).

‘Pre-exploitation’ sea otter population estimates have been informed by a diversity of sources including early maritime trade pelt landings (Nichol and others 2015) and habitat suitability models (COSEWIC 2007; Gregr and others 2008). However, the applicability of these estimates as a representation of ‘natural’ baselines across the Pacific Northwest Coast is limited. The annual number of pelts exchanged at the beginning of the maritime fur trade for example is unlikely to be a direct proxy for the number of sea otters living on the coast in a given year. Like many valuable items of ‘wealth’, sea otter pelts were collected, curated, accumulated and handed down across generations as part of family dowries well before the maritime fur trade (Sapir 1922; Drucker 1951; Uu-a-thluk 2007; Sloan and Dick 2012). Consequently, the number of sea otter pelts traded in the late 1700s has the potential to reflect decades of sea otter hunting effort prior to contact. Moreover, suitable sea otter habitat is estimated from data and observations of sea otter ecology from recent decades when humans have been precluded from hunting sea otters by federal law and centuries after epidemics greatly reduced Indigenous populations (McMillan and McKechnie 2015) and their harvesting practices, stewardship role and thus influence in coastal ecosystems. Consequently, these ‘pre-exploitation’ sea otter estimates do not incorporate the millennia of interactions between sea otters and humans dating back to the early Holocene (Fedje and others 2005; McKechnie and Wigen 2011; Szpak and others 2012).

Archaeological data, specifically shifts in abundance, size and age of faunal remains in zooarchaeological assemblages, are increasingly being used to extend population baselines and illuminate changes in food webs through deep time (Steneck and others 2004; Lotze and others 2011; Braje and others 2017). This method can be extended to investigate changes in food web interactions between humans, sea otters and invertebrate prey (Corbett and others 2008; Dunne and others 2016). Today, sea otters are known to serially reduce the size and abundance of their invertebrate prey over time where established populations reside (Estes and Palmisano 1974; Kvitek and others 1992; Salomon and others 2007). With an understanding of such size shifts in contemporary food webs, one can draw inferences on the magnitude of predation by sea otters throughout the Holocene from the size structure of ancient sea otter prey found at ancient settlements. For example, in the Aleutian Islands, the presence of sea urchins exceeding sizes that can be sustained in areas foraged by sea otters suggests that sea otter populations were kept below carrying capacity locally by human influence (Simenstad and others 1978; Corbett and others 2008). Similarly, on the Channel Islands in southern California, the presence of large red abalone and other shellfish indicates the lack of sea otter predation (Erlandson and others 2005; Erlandson and others 2008; Braje and others 2009). These findings are consistent with zooarchaeological data and isotopic analyses from British Columbia (BC) (Szpak and others 2012), and Indigenous knowledge and oral histories from the coast of BC and southeast Alaska, where communities both valued sea otters pelts as items of wealth and had incentive to limit sea otters from foraging in specific areas containing valued shellfish (Jewitt 1816; Swan 1857; Sapir 1922; Drucker 1951; Gardner 2003; Stewart 2005; Osborne 2007; Uu-a-thluk 2007; Arima and others 2009; Salomon and others 2015; Salomon and others 2018; Ibarra 2021).

On the Northwest Coast of North America, California mussels (*Mytilus californianus)* provide a prime candidate as a size-based indicator for sea otter predation. Singh and colleagues (2013) found that both mean and maximum mussel size at locations occupied by reintroduced sea otters for 20 years or more were significantly smaller than in regions where reintroduced sea otters were absent. Moreover, ancient California mussels are ubiquitous in archaeological sediments along the Northwest Coast of North America (Wessen 1988; Moss 1993; McKechnie 2014) throughout the Holocene. Their ubiquity has led to the quantification of several morphometric relationships (McKechnie and others 2015), allowing full shell length to be derived from shell fragments, which in turn enables the reconstruction of past mussel size structure (Braje and others 2018).

Here, we test the hypothesis that modern California mussel size structure is truncated by sea otter presence and occupation time. We then test the hypothesis that ancient California mussel size structure is equivalent to modern mussel size at locations without sea otters. Our results suggest that sea otters in the late Holocene were rare to absent near sites of human occupation, contrary to the general assumption that sea otters were at or near carrying capacity throughout their range prior to the Pacific maritime fur trade.

## Methods

### Historical Sea Otter Range and Contemporary Population Trends

Prior to the maritime fur trade which began in the late eighteenth century, sea otters ranged from Japan, north through the Aleutian Islands and down the Pacific coast of North America to Baja California (Barabash-Nikiforov 1947). Sea otters were ecologically extirpated from the Northwest Coast of North America by the mid-1800s (Kenyon 1969). Following over a century of their functional absence, sea otters were reintroduced to southeast Alaska, on the west coast of Vancouver Island, and to northern Washington via translocations from the Aleutian Islands between 1969 and 1972 (Kenyon 1970; Bigg and MacAskie 1978). Sea otters were documented on the Central Coast of BC in 1989 and are thought to have originated from the population reintroduced to the western Vancouver Island 20 years prior (Nichol and others 2015).

Today, sea otter population sizes and trajectories vary spatially and temporally across the Pacific Northwest Coast. Last surveyed in 2017, the south coast population of sea otters in BC, located on the west coast of Vancouver Island (*n* = 6263), was over three times that of the Central Coast population (*n* = 1838) and western Washington population (*n* = 1753) (Sato 2018; Nichol and others 2020). From 2013 to 2017, annual population growth rates of sea otters in longer occupied locations of BC’s South and Central Coasts (1.55–2.88% year ^−1^) were between 2.6 and 15.8 times lower than those in more recently occupied locations (7.52–24.51% year ^−1^). In fact, modelling efforts provide strong evidence that sea otters are experiencing negative density dependence and reaching carrying capacity in these longer occupied locations (Nichol and others 2020).

### Ecology of Sea Otters and Mussels

Dietary diversification within and among sea otter populations can be attributed to sea otter density, sex, habitat, and occupation time (Estes and others 2003; Tinker and others 2008; Newsome and others 2009; Smith and others 2021), as otters turn to a wider variety of less-valued prey items after high-value prey are diminished (Estes and others 1981; Ostfeld 1982; Laidre and Jameson 2006; Rechsteiner and others 2019). As sea otters re-occupy areas after decades of extirpation, range expansion occurs first through the expansion of bachelor males, who tend to establish new high-density rafts in previously unoccupied territory (Garshelis and others 1984), followed by smaller rafts of females, pups and territorial males, each of which exhibit unique foraging behaviours. For example, along BC’s Central Coast, at recently occupied sites, sea otter diet diversity was low but energy rich and dominated by sea urchins (*Mesocentrotus* and *Strongylocentrotus* spp.; > 60%) collected from 10 to 40 m depth. At longer occupied sites, sea otter diets became more diverse but energy poor and were dominated by clams (Veneroid; > 30%), mussels (*Mytilus* spp.; > 20%) and crab (Decapoda; > 10%) collected from shallow (< 10 m) kelp habitats (Rechsteiner and others 2019). As a less-valued prey item, mussels are typically consumed when the abundance of high-valued and energy rich prey such as sea urchins have been depleted (VanBlaricom 1988; Singh and others 2013; Rechsteiner and others 2019). Accordingly, the presence of large mussels in the rocky intertidal would indicate low to no sea otters and/or short to no occupation time.

Though many factors affect the size and growth rate of intertidal and subtidal macroinvertebrates, sea otters have been documented to exert strong top-down control resulting in reduced average sizes of their prey across a variety of habitats and environmental conditions (Estes and Palmisano 1974; Kvitek and others 1992; Fanshawe and others 2003; Salomon and others 2007; Singh and others 2013; Lee and others 2016; Burt and others 2018; Hale and others 2019). In some cases, environmental variation affecting macroinvertebrate growth rates have been found to have minimal effect in comparison with sea otter predation. For example, in Prince William Sound, Alaska, the closely related blue mussel *Mytilus trossulus (formerly M. edulis*) was smaller where sea otter occupation time was longer, and predator exclusion experiments confirmed that this difference in mussel size was due to keystone predation and not to variation in location-level environmental factors (VanBlaricom 1988).

Although top-down control by predators is a dominant ecological process structuring benthic macroinvertebrate size and spatial distribution, mussel growth has also been shown to be strongly influenced by bottom-up drivers such as pelagic primary production which can vary at scales from 10 to 100 s of kilometres (Menge 2000). This bottom-up process, mediated by upwelling events and nutrient input, has been associated with changes in secondary production including mussel growth and recruitment rates (Menge and others 1994; Menge and others 1997; Menge and others 2009). Abiotic effects such as sea surface temperature, wave exposure, aspect, slope and rugosity are also well known to effect mussel growth (Blanchette and others 2007; Menge and others 2008; Jazwa and others 2020). Importantly, it has become increasingly clear that bottom-up and top-down processes affecting mussel size interact. For example, when pelagic primary production is limiting, keystone predation by sea otters can increase the supply of kelp-derived organic carbon that can magnify filter feeder growth rates, such as those of *M. trossulus* (Duggins and others 1989).

### Experimental Design

#### Modern Locations

**Figure 1:**
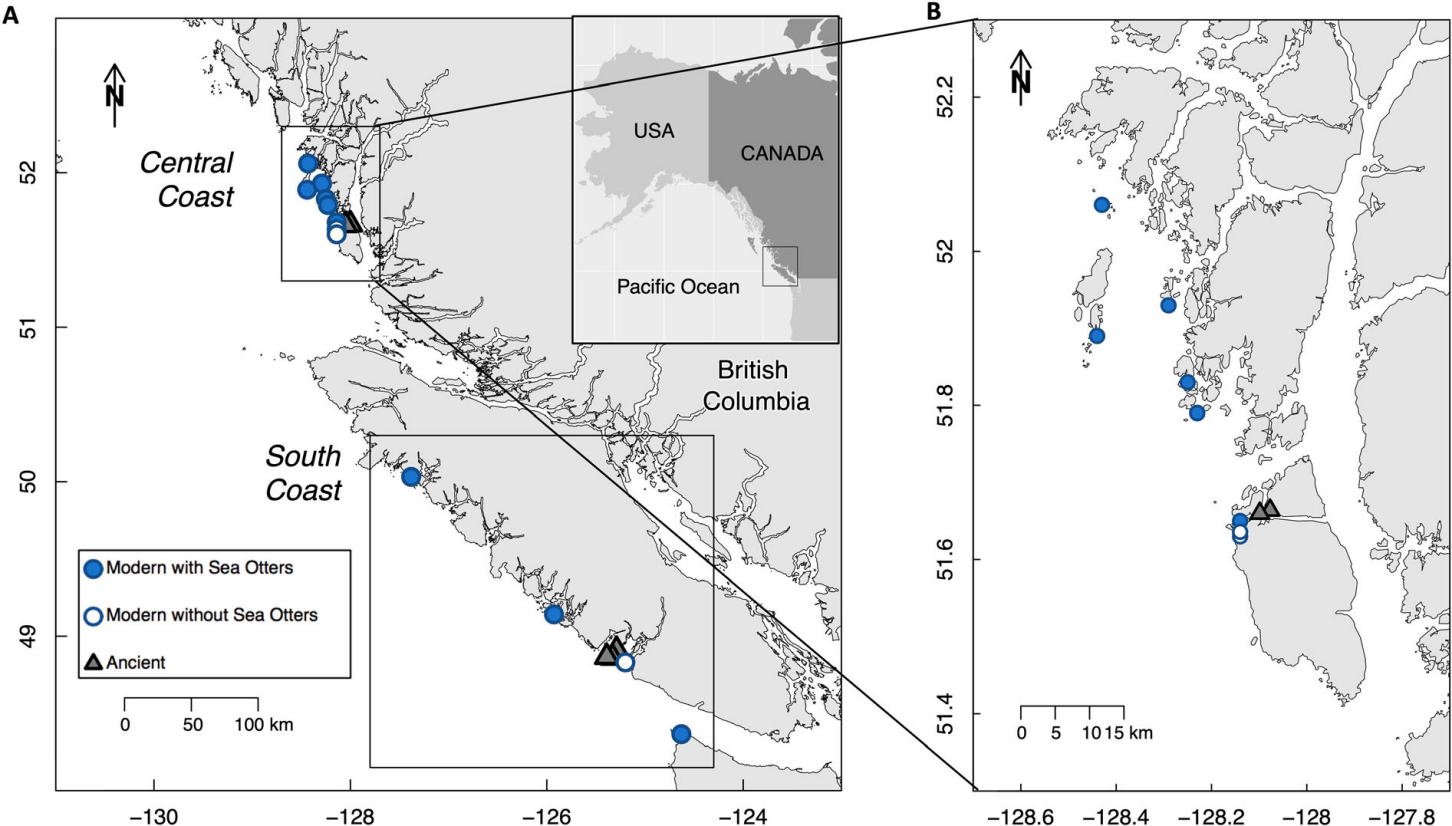
**a** Size of modern and ancient California mussels (*Mytilus californianus*) were compared within two regions of the Pacific Northwest Coast (see insets). Modern mussels were sampled from twelve intertidal locations with (filled circles) and without (unfilled circles) sea otters, including four from the South Coast (Singh and others 2013) and **b** eight locations along an established gradient of sea otter occupation time spanning 0–37 yrs from the Central Coast (this study). Ancient mussels were recovered from archaeological sites in British Columbia (*n* = 6, grey triangles).

To establish the relationship between sea otter presence and California mussel (*Mytilus californianus*) size, we measured modern mussel sizes at locations varying in sea otter occupation time on the Central Coast of British Columbia (BC), Canada. We added these data to an existing data set (Singh and others 2013) from the South Coast of BC and northern Washington, USA (Figure 1). For both data sets, locations were selected by identifying intertidal rocky reef benches that shared similar abiotic characteristics (for example, wave exposure, aspect, slope and rugosity) but varied in sea otter occupation time. We used a space-for-time substitution (Pickett 1989), whereby sea otter range expansion in space was used as a proxy for sea otter predation pressure over time. We defined sea otter occupation time as the minimum length of time a location had been observed to have been occupied by sea otters. Along the South Coast, four locations represent four temporal categories of occupation time: Kyuquot Sound, BC (40 years), Neah Bay, WA (20 years), Clayoquot Sound, BC (5 years), and Barkley Sound, BC (0 years). Along BC’s Central Coast, we measured mussels from eight locations that represent five temporal categories of occupation: (0, 4, 6–8, 21, and 37 years). In this region, where we had more detailed sea otter range expansion information, a location was considered occupied by the presence of a raft (> 3 otters) within 5.5 km of a location (Stevenson and others 2016). In both regions, raft presence was recorded during range-wide population surveys conducted every five years from 1990 to 2013, augmented by surveys and reports of sea otter rafts in between these surveys (Nichol and others 2005; Nichol and others 2009; Nichol and others 2015), and field observations (Burt and others 2018; Rechsteiner and others 2019).

In these two coastal regions, California mussels are among the most common and accessible large sessile macroinvertebrates which concentrate in the mid-intertidal, can be long-lived (> 20–50 years) and grow to large sizes (over 20 cm in length) (Seed and Suchanek 1992). Contemporary human mussel harvest consists of periodic use by coastal First Nations and non-indigenous recreational users. Harvest pressure is low, due in part to distance from contemporary communities and the prevalence of paralytic shellfish poisoning in this species which deters food and recreational harvesters (Finnis and others 2017).

#### Archaeological Sites

We examined shellfish assemblages containing abundant California mussel fragments from six archaeological sites (Figure 1) in separate ancient Indigenous villages in two regions of coastal British Columbia spanning a range of time periods from 2,700 years ago up until approximately AD 1900 on the South Coast and 6000–300 cal BP on the Central Coast (Table S1). On the South Coast, samples come from four sites within the Broken Group Island archipelago, in the territory of the modern Tseshaht First Nation on southwestern Vancouver Island. Central Coast samples come from two sites in the territories of the Haíłzaqv (Heiltsuk) and Wuikinuxv First Nations on Calvert and Hecate Islands.

Shellfish assemblages from archaeological sites were extracted using several methods, including vibracore, auger, column samples, and hand collection from eroding midden exposures. All samples were screened through 2-mm mesh and nothing below this size was measured. Further site and sampling details regarding archaeological data can be found in the supplemental material (Table S1).

### Estimating Ancient Mussel Shell Length

**Figure 2:**
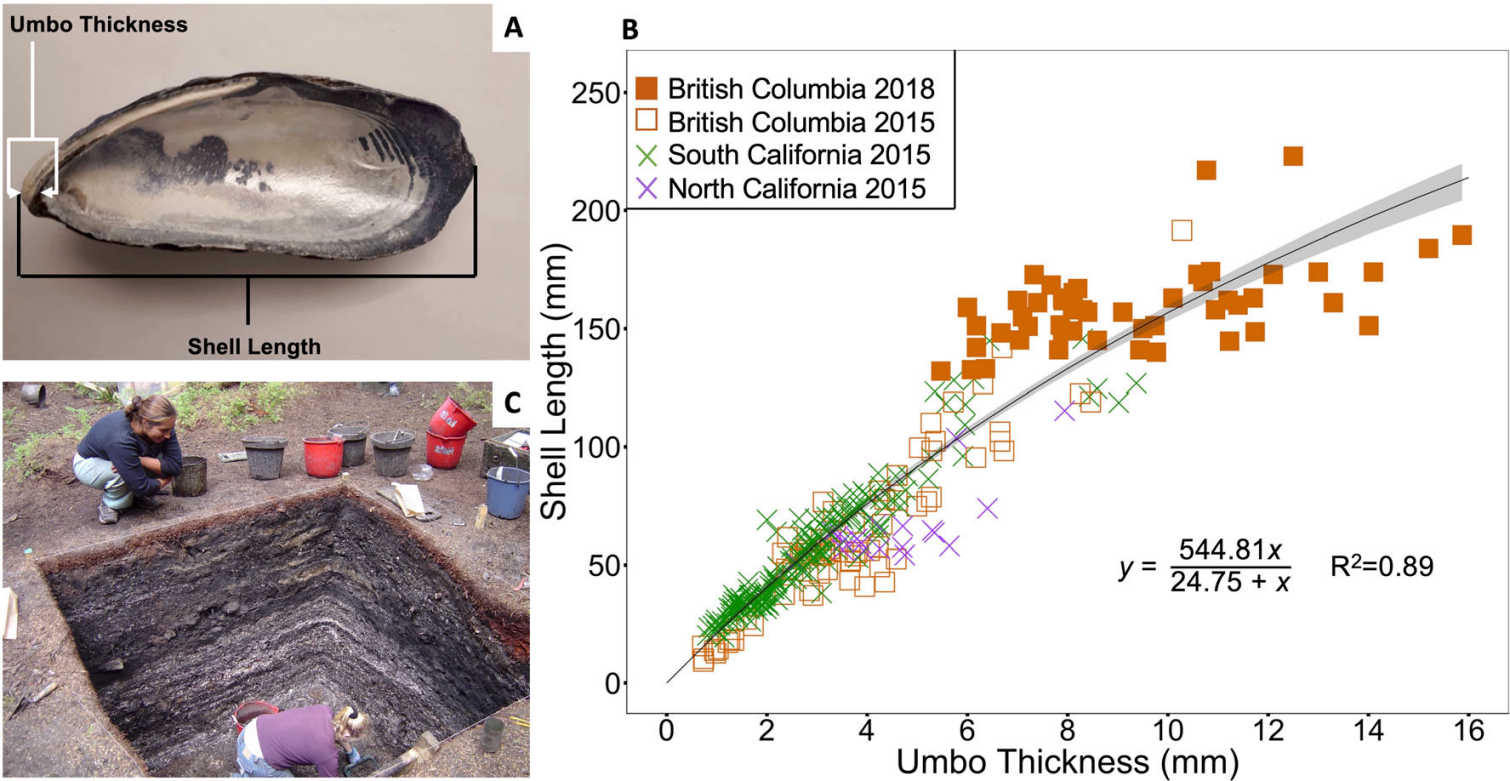
**a** Modern California mussel (*Mytilus californianus*) umbo thickness and total shell length were measured to establish **b** a saturating relationship between these two morphometrics (*n* = 313 mussels, filled orange squares from this study, all other symbols from McKechnie and others 2015). 95% confidence interval of the mean is represented by the grey band around the curve. **c** Archaeological umbos were recovered from excavation in shell midden deposits, where fragmentary umbos were measured (Photo: McKechnie), and their total shell length estimated from the relationship established in **b**.

To determine total California mussel shell length from fragmentary ancient specimens, we expanded on an existing valve length to umbo thickness relationship established for modern mussels (McKechnie and others 2015) by including large mussel shells (130–223 mm, *n* = 50) collected from Barkley Sound, BC, in 2018. This contemporary morphometric relationship was chosen to estimate ancient shell length because umbos are the most robust portion of the mussel preserved in midden deposits, and represent the origin of shell growth, a strong predictor of full shell length (Seed 1968; Ford and others 2010). Umbo thickness was measured from the tip of the umbo to the inside of the hinge using digital calipers (Figure 2a), whereas shell length was measured from the outer umbo to the point on the shell’s end that measures the longest valve dimension (for detailed methods see McKechnie and others (2015) and Singh and McKechnie (2015)). Inter-observer measurement discrepancy and error were minimized by using a consistent measurement approach, carried out by two trained observers.

We fit a saturating relationship to these data using a least squares method with the *nls()* function in R, predicting shell length from umbo thickness (Figure 2b; Eq. 1).

1 $$ y = ~\frac{{544.81x}}{{24.75 + x}} $$

This equation was used to estimate all total shell length values representing our archaeological (ancient) data. Confidence intervals were computed using Monte Carlo simulation of the normal distribution around each predictor value with the predictNLS() function in the propagate package in R.

### Modern Mussel Field Sampling

In the South Coast region, California mussels were sampled by Singh and others (2013) at three intertidal sites nested within four locations varying in sea otter occupation times. Ten 25 × 25 cm quadrats randomly placed along a transect running parallel to the shoreline, in the middle and lower regions of a mussel bed. From each quadrat, 15–50 individuals were randomly selected and measured along their longest valve dimension.

On the Central Coast of BC, we recorded California mussel size from eight intertidal locations, varying in sea otter occupation time from 0 to 37 years (Figure 1b). At each location, we measured mussels from six 25 × 25 cm quadrats randomly placed along a 30 m horizontal transect at the middle (*n* = 3) and lower (*n* = 3) regions of the mussel bed. Rather than taking a subset as Singh and others (2013) did, we collected and measured all mussels along their longest valve dimension.

### Statistical Analysis

We compiled size frequency distributions of both modern and ancient mussel size by location, and converted them to size spectra—linear models fit to size frequency data, providing a slope to describe the relationship between abundance and body size class (Edwards and others 2017). Prior to this analysis, we removed all mussel sizes below 20 mm from our data set to compare randomly sampled modern intertidal mussel populations with archaeological mussels, which include selectively harvested individuals for food as well as mussels caught as bycatch. This 20 mm cut-off was selected because it was the smallest estimated shell length from our archaeological data, representing an approximate lower detection limit for archaeological mussels. Furthermore, mussels below 20 mm are unreliably detected and measured in both modern and archaeological samples. Though 20 mm is below the size of mussels typically consumed, opportunistic examination of the size structure of a modern mussel catch by Indigenous harvesters in 2017 demonstrates that mussels in this size range are often represented in the bycatch of a mussel harvest (Figure S1, Table S2) and are occasionally represented in archaeological deposits. We grouped shell lengths into 10-mm bins to capture the range of values needed to fit a linear relationship without small-scale variability interfering with detection of the overall trend. The proportion of values in each bin was then calculated, and the midpoint of each bin was plotted against the log-transformed proportion of their abundance, converting size frequency distributions to size spectra. 95% confidence intervals for each size spectra were generated with the geom_smooth function in ggplot in R.

#### Modern vs. Ancient Mussel Size Comparison

To test for the effect of sea otter presence on the relative proportion of mussel sizes in both modern and ancient assemblages, we ran an ANCOVA with ecological and temporal context (modern mussels with sea otters, modern mussels without sea otters, and ancient mussels) as a fixed effect and mussel length as a covariate. We then ran Tukey post hoc pairwise comparisons for each ecological and temporal context to quantify the degree to which each differed from the others.

To represent the size structure of modern mussels at locations on the South Coast with sea otters, we combined modern mussel lengths from two locations with sea otter occupation times of 20 and 40 years. On the Central Coast, we combined modern mussel lengths from two locations with sea otter occupation times of 21 and 37 years. Within the South and Central Coast regions, the size structure of modern mussels at locations without sea otters is represented by modern mussel lengths from all locations, within those regions, where sea otters were absent (0 years occupation). All archaeological mussel size data from within each region were included to represent “ancient” temporal context (that is, prior to the maritime fur trade).

#### Modern Mussel Size Structure

To test for an effect of sea otter occupation time on the relative proportion of modern mussel size, we ran an ANCOVA with occupation time as a fixed effect and mussel length as a covariate. We then ran Tukey post hoc pairwise comparisons between each occupation time category.

#### Assumptions and Limitations

Comparing modern mussel sizes from randomly sampled intertidal populations to ancient mussel sizes from catch records embedded in human settlement sites has its limitations. Specifically, ancient shell middens encompass fisheries data reflecting non-random, size-selective harvest practices by people. Consequently, randomly sampled intertidal mussel populations from modern sites, both with and without sea otters, are less likely to capture the same frequency of large mussels present in ancient catch data where people were preferentially selecting large individuals, and more likely to capture small size classes. In the case of mussels however, where clumps of individuals of different size classes are often attached together with byssal threads, harvested samples may more closely resemble randomly sampled in situ populations than typical size-selective fisheries. Further comparisons of randomly sampled intertidal mussel populations and mussel catches from the same areas varying in sea otter occupation time would illuminate the magnitude of this limitation, assuming contemporary harvest practices mimic those from the Holocene (Figure S1, Table S2).

## Results

### Modern Umbo-Shell Length Regression

We established an asymptotic relationship between modern mussel umbo thickness and total shell length (Figure 2b). Specifically, umbo thickness explained 89% of the variation in total shell length (*y* = 544.81x / 24.75 + x, *R*^2^ = 0.89, *n* = 313 mussels), improving upon a linear fit (*y* = 13.3 + 14.5x, *R*^2^ = 0.87, Figure S2) and a previously published linear regression by 5% (McKechnie and others 2015; *R*^2^ = 0.84). We used this asymptotic relationship to estimate the total shell length of our archaeological mussel fragments due to its higher *R*^2^ and the more conservative length estimates it predicts for thicker umbos compared to the linear model.

### Modern vs. Ancient Mussel Size Comparison

**Figure 3:**
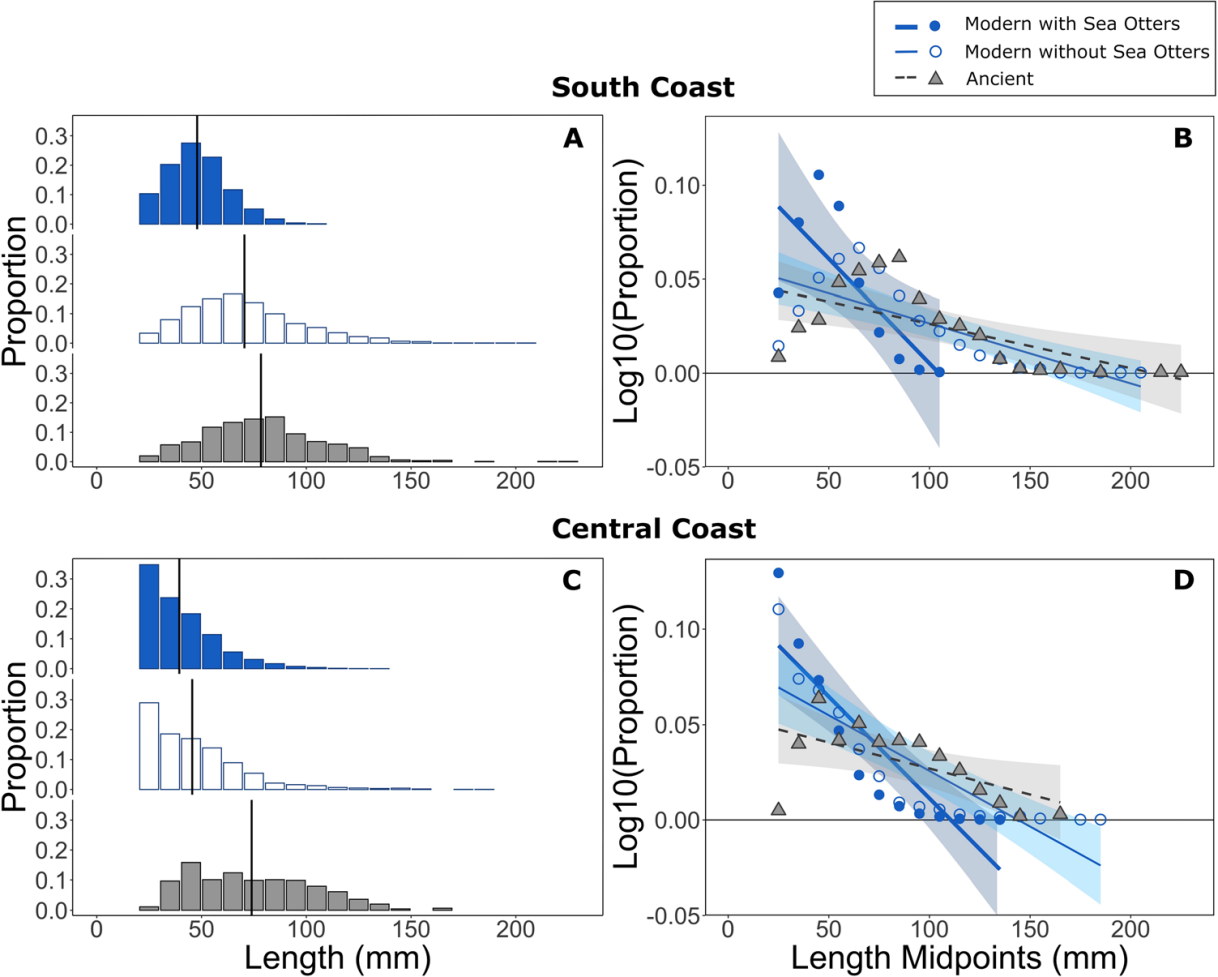
Size distributions and size spectra of California mussels (*Mytilus californianus*) from the South Coast **a**, **b** and Central Coast **c**, **d**. Modern mussel sizes at locations with sea otters present for 20–40 years (blue filled bars and circles) and without (blue unfilled bars and circles). Ancient mussel sizes (grey bars and triangles) estimated from mussel umbos collected from archaeological sites. Mean mussel sizes (vertical black lines). 95% confidence interval of the mean is represented by the coloured bands around size spectra lines. Code and measurement data presented in the supplemental material.

Overall, modern California mussels were smaller at locations with sea otters compared to locations without sea otters, on both the South and Central Coast (Figure 3). Moreover, we observed that ancient mussel size distributions most resembled modern mussel size distributions at locations without established populations of sea otters on both the South and Central Coasts (Figure 3). Specifically, along the South Coast, the size distribution of modern mussels at locations occupied by sea otters was truncated (mean = 47.91 mm ± 0.38 SE, *n* = 1421) in comparison with both the size distribution of modern mussels at locations without sea otters (mean = 70.47 mm ± 0.74 SE, *n* = 1357) and the ancient mussel size distribution (mean = 78.35 mm ± 1.00 SE, *n* = 801; Figure 3a, Table S3).

Based on our size spectra analysis of South Coast mussels, we found a significant effect of ecological and temporal context (modern with sea otters, modern without sea otters, and ancient mussels) on the relationship between mussel length and the log-transformed proportion of mussels (Table 1a; *F* = 6.17, *p* = 0.005). We detected significantly steeper mussel size spectra at modern locations with sea otters than both size spectra from modern locations without sea otters (Table 2a; *p* = 0.008) and ancient mussels (Table 2a; *p* = 0.003). In contrast, we did not detect significant differences in size spectra between modern mussels at locations without sea otters and ancient mussels (Table 2a, *p* = 0.72).

**Table 1:** Results of ANCOVAs Testing the Effect of A) Ecological and Temporal Context (Modern with Sea Otters, Modern Without Sea Otters, and Ancient Mussels) and B) Occupation Time (Years) on the Relationship Between California Mussel Length (mm) and the Log-Transformed Proportion of California Mussels on the South Coast and Central Coast

Model Region	Interaction Term	F	P
(A) Modern-Ancient			
*Log10(Proportion) of mussels* ~ *Length* + *Context* + Length*Context			
South Coast	Length*Context	6.17	0.005*
Central Coast	Length*Context	8.02	0.001*
(B) Modern			
*Log10(Proportion) of mussels* ~ *Length* + *Occupation time* + Length*Occupation time			
South Coast	Length*Occupation time	2.23	0.098
Central Coast	Length*Occupation time	13.57	1.78e–07*

Significant effects are starred.

**Table 2:** Results of Tukey’s Post Hoc Pairwise Comparisons of the Relationship Between California Mussel Length (mm) and Log-Transformed Proportion of Mussels (Slopes of Each Size Spectra), Between Each (A) Ecological and Temporal Context and (B) Occupation Time on the South Coast and Central Coast

Model Region	Pairwise Comparison	*P*
*(A) Modern-Ancient*		
Log10 (Proportion) of mussels ~ Length + Context + Length*Context		
South Coast	Modern without Sea Otters—Ancient	0.72
	Modern with Sea Otters—Ancient	0.003*
	Modern with Sea Otters—Modern without Sea Otters	0.008*
Central Coast	Modern without Sea Otters—Ancient	0.13
	Modern with Sea Otters—Ancient	0.0009*
	Modern with Sea Otters—Modern without Sea Otters	0.034*
*(B) Modern*		
Log10(Proportion) of mussels ~ Length + Occupation time + Length*Occupation time		
South Coast	0–5	0.98
	0–20	0.32
	0–40	0.20
	5–20	0.43
	5–40	0.25
	20–40	0.90
Central Coast	0–4	0.028*
	0– 6–8	0.23
	0–21	< 0.0001*
	0–37	0.42
	4– 6–8	0.90
	4–21	0.0001*
	4–37	0.71
	6– 8–21	< 0.0001*
	6– 8–37	1.0
	21–37	< 0.0001*

Significant results are starred.

We found similar patterns, although less pronounced in the Central Coast region (Figure 3c), with a smaller difference between mean mussel valve size at modern locations with sea otters (mean = 39.40 mm ± 0.22 SE, *n* = 5594) compared to modern locations without sea otters (mean = 45.56 mm ± 0.35 SE, *n* = 4488) and ancient mussel size (mean = 73.94 mm ± 1.40 SE, *n* = 436, Table S3). Maximum mussel size was smaller at modern locations with sea otters (132 mm) than at modern locations without sea otters (182 mm) and estimated ancient mussel size (166.0 mm, + 3.51 -4.13 mm 95%CI, Table S3). We also found a significant effect of ecological and temporal context on the relationship between mussel length and log-transformed proportion of mussels on the Central Coast (Table 1a; *F* = 8.02, *p* = 0.001). Again, we detected a significantly steeper mussel size spectra at modern locations with sea otters than modern locations without sea otters (Table 2a; *p* = 0.034) and ancient mussels (Table 2a; *p* = 0.0009). Lastly, we did not detect a significant difference between mussel size spectra at modern locations without sea otters compared to ancient mussels (Table 2a, *p* = 0.13). Ancient mussel size data are consistent with a 2017 Indigenous harvest of mussels collected on the South Coast in an area without otters in which mussel size ranged from 11 to 170 mm, with a mean length of 76.33 mm ± 2.18 (*n* = 261, Table S2).

### Modern Mussel Size and Sea Otter Occupation Time

**Figure 4:**
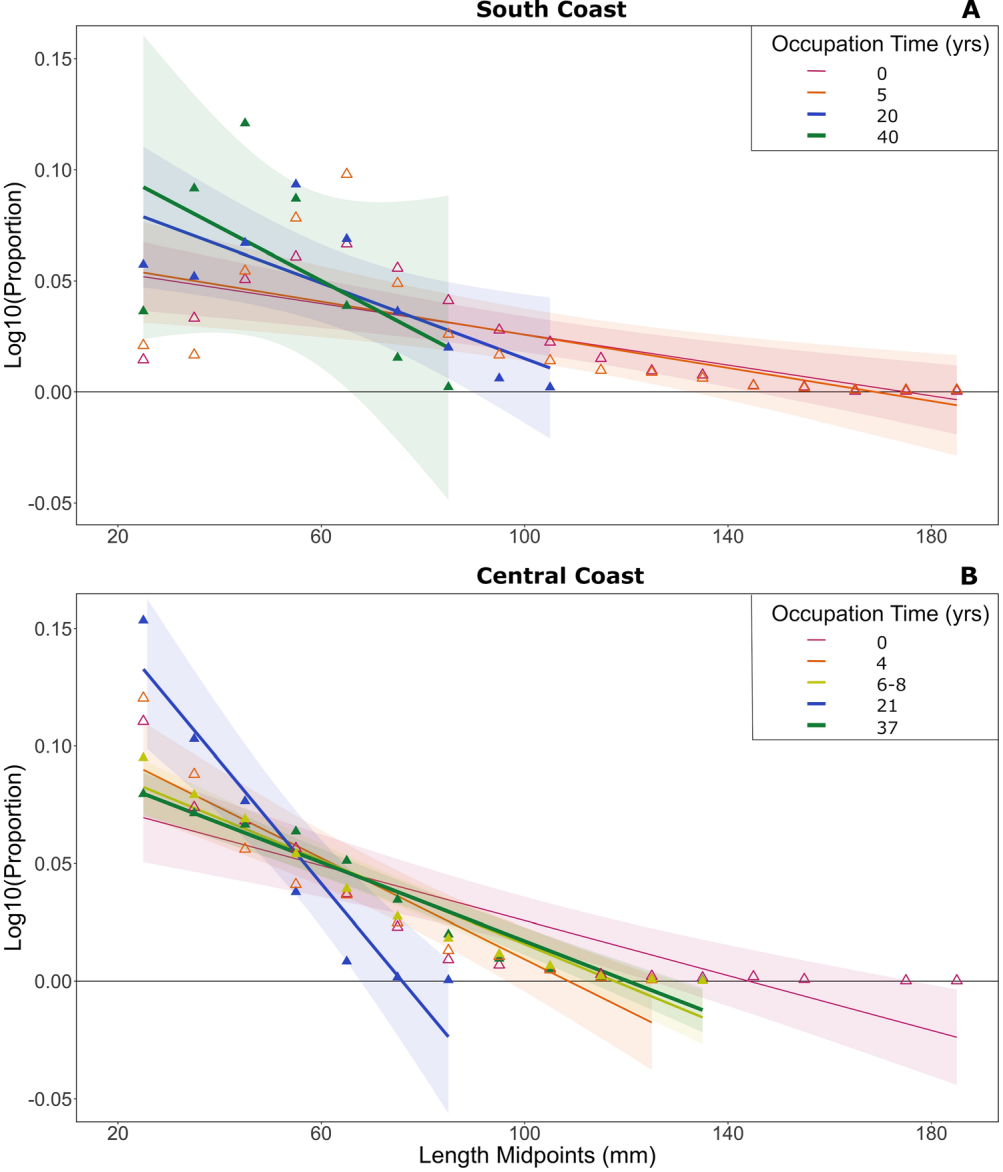
Size spectra of California mussels (*Mytilus californianus*) from all modern locations on the **a** south and **b** central portions of the Pacific Northwest Coast grouped by sea otter occupation time. Open triangles represent locations occupied by sea otters for 5 years or less, while closed triangles represent locations occupied for more than 5 years. See supplemental material for further location information. 95% confidence interval of the mean is represented by the coloured bands around size spectra lines.

On the South Coast, the slope of modern size spectra tended to become steeper with increasing sea otter occupation time (0–40 years occupation; Figure 4, Figure S3). Building on Singh and others (2013), this trend is consistent with the observation that larger mussels are more common at locations where otters are absent and get smaller as otter occupation time increases (Figure S3). However, this effect of occupation time on the relationship between mussel length and the log-transformed proportion of mussels was not significant (Table 1b; *F* = 2.23, *p* = 0.098), and pairwise comparisons of these slopes at each occupation time revealed no significant differences (Table 2b, Table S4).

On the Central Coast, where we have more finely resolved spatially explicit information on modern sea otter occupation time, greater mussel sampling effort and a greater number of small mussel sizes, we detected a significant effect of occupation time on the relationship between mussel length and the log-transformed proportion of mussels (Table 1b; *F* = 13.57, *p* = 1.78e-07). Modern locations with sea otters tended to have steeper size spectra than locations without sea otters. However, in contrast to the South Coast, we did not observe a consistent increase in slope with each increase in occupation time (Figure 4, Figure S3). The shallowest slope of size spectra from our modern central coast data was found where otters were absent (occupation time = 0 years; slope = -0.00058). However, the steepest slope value from our length-proportion size spectra was found at 21 years of occupation (slope = -0.0026), and the slope at our longest-occupied location was shallower (37 years; slope = -0.00084, Table S4). Through pairwise comparisons, we found that the mussel size spectra at 21 years of occupation varied significantly from all other locations (Table 2b, *p* ≤ 0.0001). On the Central Coast, we also detected a significant difference between the mussel size spectra at locations that have experienced four years of sea otter occupation compared to locations where sea otters have yet to re-occupy (Table 2b, *p* = 0.028).

## Discussion

Our results provide further evidence supporting the hypothesis that sea otters existed below carrying capacity in proximity to human settlements during the late Holocene on the Northwest Coast of North America (Simenstad and others 1978; Erlandson and others 2005; Corbett and others 2008; Erlandson and others 2008; Szpak and others 2012). That is, Indigenous communities, prior to the maritime fur trade, maintained access to significantly larger mussels than those found at modern locations with sea otters (Figure 3, Table 2a). We suggest that these collective findings are indicative of human-mediated limitation of sea otters and their predatory effects on shellfish where humans persistently harvested large macroinvertebrates.

Along contemporary rocky shorelines, we found that the relationship between sea otter occupation time and modern mussel size varied within and between regions. Mussel size distributions became increasingly truncated with sea otter occupation time across our study region, with size spectra varying significantly with sea otter occupation time on the Central Coast, but not the South Coast (Figure 4, Table 1b,2b). Collectively, our comparison of modern mussel sizes with archaeological data extending well beyond the time scales of modern observations, has expanded our understanding of how sea otters persisted in the context of human occupation of the Pacific Northwest Coast prior to the maritime fur trade.

### Large Ancient Mussel Sizes Reflect Low Sea Otter Predation in Ancient Times

Our results suggest that the effect of sea otter predation on mussel size was limited in proximity to human settlements where mussels were regularly harvested during the late Holocene (Figure 3). Large mussels between 120 and 220 mm existed among the faunal remains at ancient settlement sites within two regions of the Pacific Northwest Coast and were virtually absent from modern sites occupied by sea otters in these same regions today. Although the larger mussels found in our archaeological assemblages may reflect a bias towards the collection of larger mussels in the field by humans, the presence of these large mussels can only occur if they are available to harvest and have not been eliminated by sea otter foraging. Given that our samples of ancient mussels come from fisheries catch data reflecting human harvest effort, it is additionally possible that human foraging pressure reduced mussel sizes found in middens through time, via both size selectivity and resource depression (Botkin 1980; Erlandson and others 2008; Braje and others 2018). If true, mussel sizes in the absence of both sea otters and humans could have been even larger.

Our results are broadly consistent with archaeological investigations of faunal assemblages elsewhere in the northeastern Pacific Rim. Analyses of shell middens from Alaska and California reveal widespread abundances of large-sized macroinvertebrates (Simenstad and others 1978; Moss 1993; Corbett and others 2008; Erlandson and others 2008; Dunne and others 2016) indicating that sea otters may have been absent or well below carrying capacity from a variety of coastal areas with substantial Indigenous settlement histories. We surmise that Indigenous peoples reduced sea otter abundance near valued shellfish harvesting and mariculture sites through a combination of five complementary mechanisms: (1) direct mortality via hunting, (2) indirect, nonlethal, unintentional exclusion via learned antipredator behavioural response by sea otters to hunting, (3) exploitative competition between sea otters and humans for shared shellfish prey, (4) emigration by sea otters away from human settlement sites to productive kelp forest habitats with more energetically profitable prey and (5) indirect, nonlethal, intentional exclusion via fear-based behavioural responses to human disturbances designed to minimize sea otter impacts on local food sources. All of these potential sea otter exclusion pathways would have operated through traditional spatially explicit marine tenure systems and associated governance protocols and management practices (Szpak and others 2012; Salomon and others 2018). We recognize the challenge of disentangling these mechanisms but note the relevance of contemporary traditional knowledge holders in illuminating the latter mechanism which we discuss below.

### Modern Mussel Size Spectra and Trophic Dynamics

Our investigation of modern mussel size spectra (Figure 4) suggests that the effect of sea otter occupation time on mussel size is variable. Although locations on the South Coast show a clear trend of decreasing mussel size as sea otter occupation time increases, this pattern is consistent only to a point on the Central Coast. Although 21 years of sea otter occupation on the Central Coast was associated with the smallest reported mean mussel size, our longest occupied location (37 years) had larger mussels than expected. We controlled for abiotic factors such as wave exposure, aspect, slope and rugosity across locations, all known to affect mussel size; consequently, additional variables may be at play and explain this result.

The degree to which sea otter occupation time can predict the size structure of its prey varies as a function of predator population density, rate of range expansion, predator–prey selectivity, and prey capture rates, each of which are affected by factors such as prey quantity, quality, and productivity, as well as habitat diversity and shoreline complexity (Hoyt 2015; Hessing-Lewis and others 2018; Hale and others 2019; Smith and others 2021). Comparatively, the South Coast region contains five times the sea otter population on a more linear coastline relative to the Central Coast region which encompasses a greater multitude of small islands, deep fjords, and channels. It is possible that mussels at the Central Coast’s oldest sea otter re-occupation location are experiencing a recovery following size depression by sea otter rafts that have moved on given the close proximity of diverse suitable foraging habitats that exist elsewhere along this highly involuted coastline. Intertidal mussel growth rates may simultaneously be enhanced by an increase in kelp-derived organic carbon stemming from the cascading effects on sea otters leading to deeper and larger subtidal kelp beds at long occupation sites. Alternatively, the population density of sea otters at this older occupation site may have never been numerically high enough to have had a significant effect on mussel size structure.

### Indigenous Management, Resource Diversity, and Resilience

Although direct human predation of marine mammals is a well-recognized factor influencing nearshore ecosystems across the Pacific Rim (Rick and others 2011; Dunne and others 2016), a growing body of archaeological, ethnographic, and historical ecological evidence demonstrates that Indigenous communities maintained deliberate systems of marine resource management prior to European contact (Rick and Erlandson 2009; Moss, 2011; Lepofsky and Caldwell 2013; Mathews and Turner 2017). Although our mussel size structure data alone does not provide direct evidence of intentional reduction of sea otters by ancient humans for the purposes of increasing shellfish production, when considered alongside ethnographic data on sea otter hunting and shellfish management practices, plus the need to secure access to reliable local sources of food (Sapir 1922; Drucker 1951; Gardner 2003; Salomon and others 2018; Ibarra 2021), our findings provide further support that human communities had incentive to actively manage their relationship with sea otters in part to enhance the productivity of key intertidal food resources along stretches of the Pacific Northwest Coast.

Specifically, a range of oral histories and ethnographies from coastal British Columbia and Alaska emphasize that in the millennia preceding the maritime fur trade, ancestral laws and governance protocols were in place to maintain the persistence of marine resources broadly and sea otters specifically. This included the proprietorship of discrete ocean spaces by hereditary Chiefs who held exclusive access rights and responsibilities over defined territories (Arima and others 2009; Trosper 2009; Kii’iljuus and Borserio 2011; Salomon and others 2018; Salomon and others 2020; Ibarra 2021). These hereditary rights and proprietorship were described as being contingent on management that sustained productive resources and their equitable distribution among community members via the potlach system of governance (Trosper 2009). Specific management practices likely developed from centuries of observing feedbacks between human impacts and environmental responses (Turner and Berkes 2006). Consistent with other studies (Erlandson and others 2005; Corbett and others 2008; Szpak and others 2012), our findings indicate support for a human-induced spatial mosaic of ecosystem states associated with the persistence of sea otter populations at locations more distant from Indigenous settlements and sea otter exclusion in proximity to regularly utilized shellfish harvesting sites. On rocky subtidal reefs, persistent sea otter populations and mature ‘old-growth’ kelp forest ecosystems encompassing a diversity of perennial kelps would have been interspersed with sea otter free urchin barren ecosystems more intensively managed for shellfish with smaller, shallower fringing kelp forests encompassing more disturbance tolerant annual kelps. This mosaic in ecosystem states would have contributed to spatial variation in the local productivity and persistence of coastal shellfish, kelp-associated reef fish and sea otter populations, while elevating biodiversity and system-wide resilience regionally.

Although our archaeological observations from only two regions limits our ability to make broadly generalizable predictions about ancient Indigenous mussel harvest size and sea otter prevalence across the Pacific Northwest Coast, we suggest that further investigations of ancient mussel size spectra and their spatial and temporal variability will provide refined insight into the scale and resolution required to fully demonstrate the likelihood and persistence of ancient spatial mosaics in sea otter abundance and ecosystem states we hypothesize here.

### Implications of Long-Term Data in Identifying Human-Mediated Ecosystem Interactions

With evidence of the profound alterations humans have made to marine ecosystems globally over the past few centuries (Jackson and others 2001; McCauley and others 2015), it has become clear that establishing “natural” baselines requires extending our investigations beyond the most recent centuries and broadening our view of what constitutes an ecosystem’s ‘natural’ state. Increasing evidence suggests that people have been integral components of ecosystems for millennia and that context-dependent interactions create spatial and temporal variation in human influenced environments (Dunne and others 2016). Insights from zooarchaeological and palaeoecological research hold considerable potential to refining our understanding of long-term human influence and environmental change on the dynamics and feedbacks within coupled social-ecological systems through time and across space.

Only by integrating archaeological, anthropological, paleobiological and ecological perspectives can the millennia of co-evolution of human and natural systems and their context-dependence be revealed (Fitzhugh and others 2019). Here, we propose that in the mid-to-late Holocene, thirteen thousand years after the extinction of terrestrial Pleistocene megafauna, humans intentionally excluded sea otters to maintain access to productive shellfish beds close to village settlements. After millennia of observation, experimentation, learning and adapting, ethnographies and TEK suggest that Indigenous stewardship practices developed to monitor and exclude predators where they were a threat to food security. Although debate over intentionality in resource conservation still lingers, there is broad consensus, particularly among social scientists studying cultural systems on the Pacific Northwest Coast, that communities intentionally developed highly structured resource management and conservation strategies to maximize community sustainability (Trosper 2009; Campbell and Butler 2010; Moss 2011; Mathews and Turner 2017).

For example, evidence of millennia-old clam gardens on the coast of BC shows that clam productivity was doubled by intentional habitat alteration and tending practices by coastal Indigenous peoples (Groesbeck and others 2014; Lepofsky and others 2021). As indicated by our Indigenous colleagues, these monumental intertidal rock-walled features and the labour invested to build and maintain them were likely safeguarded from sea otter predation (Kii'iljuus cited in Salomon and others 2018), and other benthic predators such as racoon, mink, river otters, diving ducks and geese (Kwaxistalla cited in Deur and others 2015).

### Implications for Contemporary Management and Conservation

In the case of sea otter management along the Pacific coast of North America today, the conventional method for estimating expected regional carrying capacity is to calculate the density of individuals that could persist given the total suitable habitat in the region, with the assumption that sea otters would occur at carrying capacity everywhere within their fundamental niche (Laidre and others 2002; Burn and others 2003; Gregr and others 2008). These estimates likely contribute to what has been considered a “small” recovering population when justifying the reasoning for sea otters’ current listing as a species of 'Special Concern' in Canada (COSEWIC 2007). However, our results, in addition to other lines of evidence, suggest the realized niche of sea otters on the Pacific Northwest Coast was likely smaller than is commonly considered, while the scale and distribution of coastal Indigenous settlement was more extensive than commonly considered. Moreover, the use of ocean going dugout canoes capable of travel over 40 km per day (Ames 2002) indicates that few if any coastal habitats were entirely isolated from people during the late Holocene. And yet sea otter faunal remains persist through time in the archaeological record with no evidence of regional extinctions and over ten millennia of coexistence between humans, sea otters and shellfish (Fedje and others 2005; Cannon and others 2008; McKechnie and Wigen 2011; Szpak and others 2012; Orchard and Szpak 2015). Although further archaeological research is needed to better understand variability in Indigenous settlement and resource use, our observations suggest it is likely that late Holocene sea otter populations persisted well below carrying capacity in many places throughout its range and coexisted with humans in a considerably reduced niche space.

A fundamental paradigm shift in natural resource management and conservation science is well underway where humans are increasingly being recognized as active components of linked social-ecological systems, rather than external disruptors to otherwise pristine ecosystems (Berkes and Folke 1998; Jackson and others 2001; Carpenter and others 2009; Gelcich and others 2010; Singh and others 2021). Yet the lingering assumption that sea otters once existed at carrying capacity throughout its range in the absence of human intervention continues to have real-world consequences. Currently in Canada, the federal government asserts exclusive decision-making authority over the conservation status and management of sea otters and limits hunting based on the most current coastwide population estimate, one-half the maximum population growth rate, and a recovery factor for a species listed as a *Special Concern.* None of these estimates consider humans as components of functioning coastal ecosystems. In addition, no policy tool exists to enable sea otter hunting for the purpose of reducing their negative effect on shellfish. Consequently, federal policies are currently constraining Indigenous rights and responsibility to manage their long-term relationship with this keystone predator and the coastal ecosystems in which they both are embedded. By broadening the chronological horizon, social-ecological lens and sources of data with which we assess species recovery, set restoration goals and envision conservation and management policies to meet them, the more likely we will be able to navigate towards ecologically sustainable and socially just operating space (Raworth 2012) for coastal social-ecological systems across the Pacific coast of North America.

## Conclusion

Our results challenge the widely held assumption, be it implicit or explicit, that sea otter populations were thriving at or near carrying capacity in every suitable habitat across the extent of their range before their extirpation by the Pacific maritime fur trade. Rather, sea otters appear to have been rare or absent near ancient human settlements. Away from village sites, sea otters persisted at levels, high enough to sustain hunting by people throughout the Holocene. The strong spatial variation of this remarkable keystone species and the hyperkeystone role of humans (Worm and Paine 2016) would have led to a spatial mosaic in kelp and sea urchin-dominated reefs at regional spatial scales, with widespread social-ecological consequences. Although other predators including killer whales, sharks, and even coastal wolves have been implicated in limiting sea otter populations (Estes and others 1998; Tinker and others 2016; Roffler and others 2021), the long-term role of people has not been similarly considered.

The implications of this emerging view of long-term human engagement in marine ecosystems, for archaeology, marine ecology, and natural resource policy, are profound. First, highly productive kelp forests, hypothesized to have supported coastal travel and migration routes for peoples across the Pacific Rim (Erlandson and others 2015), may have been more spatially discontinuous throughout the Holocene, serving more as ‘hops’ than a continuous ‘highway’. Second, the keystone role of sea otters, typically assumed to be universally present, would have been absent from many coastal food webs, adding considerable spatial variability to the trophic dynamics, primary and secondary production, and carbon flux assumed to exist across North America’s coastal ocean ecosystems throughout the Holocene. Lastly, this emerging understanding of the functional role of humans as components of coastal ecosystems challenges our view of ‘natural’ baselines (Dayton and others 1998; Ellis and others 2021) and our perceptions of what constitutes ‘recovered’ populations. The ghosts of ecosystems past along North America’s Pacific coastline includes humans and their role as both predators and intentional stewards.

## Supplementary Information

Below is the link to the electronic supplementary material.

Supplementary file1 (DOCX 4943 KB)

Supplementary file2 (XLSX 1038 KB)

Supplementary file3 (RMD 68 KB)
