# Supplementary material for: Archaeological and Contemporary Evidence Indicates Low Sea Otter Prevalence on the Pacific Northwest Coast During the Late Holocene
Source: Ecosystems. 2021 Aug 17;25(3):548–66. doi: 10.1007/s10021-021-00671-3 (PMC9016008; doi:10.1007/s10021-021-00671-3)
Supplement: Supplementary file 1 — Supplementary file1 (DOCX 4943 KB) [file 10021_2021_671_MOESM1_ESM.docx]

Supplemental Materials for:

Archaeological and contemporary evidence indicates low sea otter prevalence on the Pacific Northwest Coast during the late Holocene

Erin Slade, Iain McKechnie^1^* and Anne K. Salomon^2^*

^*^ Joint corresponding authors

^1^ Department of Anthropology, University of Victoria, Cornett B246a, 3800 Finnerty Rd., Victoria, BC V8P 5C2 Canada Email: iim@uvic.ca Phone: 250-721-7351 <https://orcid.org/0000-0002-4989-2711>

^2^ School of Resource & Environmental Management, Simon Fraser University, Burnaby, BC V5A 1S6 Canada Email: [Anne.Salomon@sfu.ca](mailto:Anne.Salomon@sfu.ca), Phone: 778-782-8739

**Methods**

Archaeological sites, sampling methods and dates

Table S1. Archaeological mussel umbos from collections on the (A) south and (B) central regions of the Pacific Northwest Coast, compiled by region and sampling effort.

| Site | Site/unit number | Core | Screen Size | Sample type | n | Site Date Range (yrs cal BP)* |
| --- | --- | --- | --- | --- | --- | --- |
| **(A) South Coast** | | | | | | |
| Keith Island | 306T7C |  | >6.35mm | Column Sample | 69 | 500-100 |
|  | 306T8C |  | >6.35mm | Column Sample | 44 | 800-200 |
|  | 306T8D |  | >6.35mm | Column Sample | 11 | 800-200 |
|  | 306T10A |  | >2mm | Vibracore | 11 | 1180-500 |
|  | 306T9A |  | >2mm | Vibracore | 46 | 1500-200 |
| Nettle Island | 305T | 1 | >2mm | Vibracore | 76 | 2700-200 |
| Lower Dicebox | 83T11A |  | >2mm | Auger | 42 | 800-150 |
|  | 83T6A |  | >2mm | Auger | 92 | 800-150 |
|  | 83T7C |  | >2mm | Auger | 81 | 500-150 |
| Wouwer | 206T12D |  | >2mm | Auger | 69 | 2500-1200 |
|  | 206T19B |  | >2mm | Auger | 69 | 2500-1300 |
|  | 206T20B |  | >2mm | Auger | 74 | 800-150 |
| **(B) Central Coast** | | | | | | |
| Hecate Island | EjTa-13 |  | >6.35mm | Hand collection | 62 | 6,000 to 300 |
| Calvert Island | EjTa-4 |  | >6.35mm | Hand collection | 177 | 6,000 to 300 |
| Hecate Island | EjTa-13 | VC 7 | >2mm | Vibracore | 18 | 3,370-390 |
|  | EjTa-13 | VC 5 | >2mm | Vibracore | 17 | 5,830-380 |
|  | EjTa-13 | VC 4 | >2mm | Vibracore | 75 | 5,650-490 |
|  | EjTa-13 | VC 1 | >2mm | Vibracore | 1 | 3,450-3,390 |
|  | EjTa-13 | VC 1 | >2mm | Vibracore | 4 | 4,500-3,380 |
|  | EjTa-13 | VC 2 | >2mm | Vibracore | 54 | Not dated |

*Chronological data obtained from McKechnie and others (2015; 2019), Hillis and others (2020), and Duffield and others (2017, 2018; 2020).

Investigating limitations in comparing modern and ancient mussel sizes

We opportunistically measured a random subset of unprocessed California mussels in 2017 that had been harvested for consumption by members of the Tseshaht First Nation Beachkeepers who patrol the Broken Group Islands in Barkley Sound on the west coast of Vancouver Island, BC within the South Coast region of the Pacific Northwest Coast (Figure 1A). Barkley Sound is currently unoccupied by sea otters. We collected a random subset from the bin of all harvested mussels by closing our eyes and extracting aggregated clumps of mussels until we filled two smaller buckets (n=261). After measuring the longest linear dimension of all mussels from this subset with calipers, we compiled mussel length data to produce a size-frequency distribution (Figure S1) and calculate median, mean, min and max mussel sizes (Table S2). When compared to randomly sampled intertidal mussel populations from the same South Coast region unoccupied by sea otters, we found that that the maximum mussel size was in fact greater in the randomly sampled intertidal population (max = 206 mm) than the harvested population (max = 170mm), although the sample sizes varied considerably (Table S2).

Figure S1. Size-frequency distribution of modern mussel length (mm) from California mussels collected by Tseshaht First Nation BeachKeepers during a single harvesting event at Gilbert Island, BC in July, 2017 (Slade, 2019).

Table S2. Summary of sample location information and associated mussel length arranged by region and by occupation time (for modern samples). Smallest values are underlined, while largest values are bold. Note: occupation time (years) recorded from date of collection.

| Location | Lat / Long | Sea Otter Occupation Time (yrs)* | N | Min (mm) | Max (mm) | Median (mm) | Mean (mm) |
| --- | --- | --- | --- | --- | --- | --- | --- |
| **A) Modern – South Coast** | | | | | | | |
| Barkley Sound, BC | 48.83, -125.20 | 0 | 1357 | 20 | 206 | 67 | 71 |
| Clayoquot Sound, BC | 41.14, -125.93 | 5 | 486 | 20 | 181 | 63 | 67 |
| Neah Bay, WA | 48.36, -124.63 | 20 | 425 | 20 | 103 | 53 | 52 |
| Kyuquot Sound, BC | 50.01, -127.42 | 40 | 996 | 20 | 87 | 46 | 46 |
| **B) Modern – Central Coast** | | | | | | | |
| 7^th^ Beach | 51.64, -128.15 | 0 | 4488 | 20 | 182 | 41 | 46 |
| 8^th^ Beach | 51.64, -128.15 |  |  |  |  |  |  |
| 2^nd^ Beach | 51.65, -128.15 | 4 | 1340 | 20 | 125 | 37 | 44 |
| Triquet | 51.80, -128.24 | 6-8 | 4968 | 20 | 132 | 42 | 47 |
| Spider | 51.84, -128.25 |  |  |  |  |  |  |
| Simmonds | 51.93, -128.24 |  |  |  |  |  |  |
| McMullins | 52.07, -128.41 | 21 | 3676 | 20 | 88 | 32 | 34 |
| Gosling | 58.90, -128.45 | 37 | 1918 | 20 | 132 | 47 | 49 |
| **C) Ancient – South Coast** | | | | | | | |
| Keith Island | 48.91, -125.29 | -- | 281 | 29 | 220 | 78 | 83 |
| Nettle Island | 48.93, -125.24 | -- | 94 | 23 | 133 | 51 | 54 |
| Wouwer Island | 48.86, -125.36 | -- | 211 | 20 | 141 | 77 | 79 |
| Dicebox Island | 48.86, -125.33 | -- | 215 | 22 | 185 | 82 | 83 |
| **D) Ancient – Central Coast** | | | | | | | |
| Calvert Island | 51.66, -128.08 | -- | 436 | 25 | 166 | 71 | 74 |
| Hecate Island | 51.66, -128.10 |  |  |  |  |  |  |
| **E) Modern – South Coast** | | | | | | | |
| Tseshaht Beachkeepers Mussel Harvest Data  Barkley Sound, BC | 48.87, -125.32 | 0 | 261 | 11 | 170 | 74 | 76 |

* (Burt and others, 2018; Rechsteiner and others, 2019; Nichol and others, 2020)

Results

Limitations estimating mussel length and identifying species

Although our asymptotic relationship predicting total shell length from umbo thickness captures 89% of the variation in total mussel length, this predictability varies across umbo thickness values, with greater uncertainty at a larger umbo thickness (manuscript Figure 2). However, this asymptotic relationship provides more conservative estimates of mussel length at large umbo thickness values compared to a linear fit (Figure S2). Further sampling of modern California mussels across their range of size and in varying growth conditions may decrease the uncertainty associated with mussel length predictions. Lastly, at small mussel sizes, it is difficult to distinguish between *M. californianus* and the morphologically similar blue mussel *Mytilus trossulus*, found within the same geographic range as *M. californianus,* typically in less wave exposed habitats. Though it is possible that some of our smaller measurements were *M. trossulus,* Singh and McKechnie (2015) found that the relationship between umbo thickness and total shell length is similar between the two species.

**Figure S2. Linear regression of total shell length as a function of umbo thickness (n=313 mussels). Filled orange squares were collected for this study, all other symbols are from McKechnie et al (2015). 95% confidence interval of the response mean is represented by the pink band around the regression line.**





Figure S3. Modern mussel size (A,C) and slope of size spectra (B,D) grouped by occupation time of sea otters at each location sampled on both the A,B) south and C,D) central regions of the Pacific Northwest Coast.

Table S3. Linear regression coefficients and goodness of fit for fitted size spectra predicting log(proportion) from mussel length midpoints. Values are grouped by time period and sea otter presence (Fig. 3).

| **Occupation time/period** | | **Slope** | **Y-int** | **R^2^** | **Mean length (mm)** | **± SE** | **Max length (mm)** | **Median length (mm)** | **± SE** | | | |  |
| --- | --- | --- | --- | --- | --- | --- | --- | --- | --- | --- | --- | --- | --- |
|  | **A) South Coast** | | | | | | | | | |  | | |
| Modern without sea otters | | -0.00032 | 0.058 | 0.61 | 70.47 | 0.74 | 206.0 | 66.70 | 0.74 | | | |  |
| Modern with sea otters | | -0.0011 | 0.12 | 0.59 | 47.91 | 0.38 | 103.0 | 47.01 | 0.38 | | | |  |
| Ancient | | -0.00024 | 0.050 | 0.42 | 78.35 | 1.00 | 220.0 | 76.00 | 1.00 | | | |  |
|  | **B) Central Coast** | | | | | | | | |  | |  |  |
| Modern without sea otters | | -0.00058 | 0.084 | 0.72 | 45.56 | 0.35 | 182.0 | 41.00 | 0.35 | | | |  |
| Modern with sea otters | | -0.0011 | 0.12 | 0.78 | 39.40 | 0.22 | 132.0 | 35.00 | 0.23 | | | |  |
| Ancient | | -0.00027 | 0.054 | 0.36 | 73.94 | 1.40 | 166.0 | 70.50 | 1.40 | | | |  |

Table S4. Summary of linear regression statistics for all modern mussel size spectra, arranged by occupation time on the south (A) and central (B) regions of the Pacific Northwest Coast (Fig. 4). For each region, largest slope is bold, while smallest slope is underlined. Note: occupation time (years) recorded from date of collection.

| **Location** | **Sea Otter Occupation time (yrs)*** | **N** | **Slope** | **Y-intercept** | **R^2^** | |
| --- | --- | --- | --- | --- | --- | --- |
| **A) South Coast** | | | | | |  |
| Barkley Sound | 0 | 1357 | -0.00035 | 0.06 | 0.57 | |
| Clayoquot Sound | 5 | 486 | -0.00037 | 0.063 | 0.42 | |
| Neah Bay, WA | 20 | 425 | -0.00085 | 0.1 | 0.57 | |
| Kyuquot Sound | 40 | 996 | -0.0012 | 0.12 | 0.35 | |
| **B) Central Coast** | | | | | |  |
| 7^th^ and 8^th^ Beach | 0 | 4488 | -0.00058 | 0.084 | 0.72 | |
| 2^nd^ Beach | 4 | 1340 | -0.0011 | 0.12 | 0.85 | |
| Simmonds, Spider, Triquet | 6-8 | 4968 | -0.00089 | 0.11 | 0.93 | |
| McMullins | 21 | 3676 | -0.0026 | 0.20 | 0.92 | |
| Gosling | 37 | 1918 | -0.00084 | 0.10 | 0.94 | |

* (Burt and others, 2018; Rechsteiner and others, 2019; Nichol and others, 2020)

References

Burt JM, Tinker MT, Okamoto DK, Demes KW, Holmes K, Salomon AK. 2018. Sudden collapse of a mesopredator reveals its complementary role in mediating rocky reef regime shifts. Proceedings of the Royal Society of London B 285: 20180553 DOI:10.1098/rspb.2018.0553

Duffield S. 2017. Long-term use of fish and shellfish resources revealed through vibracore sampling at EjTa-13, Hecate Island, Central Coast, BC. MA Thesis Department of Anthropology: University of Victoria.

Duffield S. 2018. Vibracore Sampling and Zooarchaeological Analysis of Fish Remains from Tl’ihuuw’a (305T) and Kakmakimilh (306T) in the Broken Group Islands, July 2017. McKechnie I, St. Claire D editors. Kakmakimilh - (306T) - Keith Island 2017/2018 Archaeological Field Program Report: Submitted to Tseshaht First Nation and Pacific Rim National Park Reserve March 7, 2019.

Duffield S, McKechnie I, St. Claire DE, McLaren D. 2020. Vibracore Sampling in the Broken Group Islands. The Midden 5: 34–37

Hillis D, McKechnie I, Guiry E, St. Claire DE, Darimont CT. 2020. Ancient Dog Diets on the Pacific Northwest Coast: Zooarchaeological and Stable Isotope Modelling Evidence from Tseshaht Territory and Beyond. Scientific Reports 10: 15630 10.1038/s41598-020-71574-x

McKechnie I. 2015. Indigenous Oral History and Settlement Archaeology in Barkley Sound, Western Vancouver Island. BC Studies 187: 191–225

McKechnie I, Singh GG, Braje TJ, Campbell B. 2015. Measuring *Mytilus californianus*: an Addendum to Campbell and Braje (2015) and Singh and McKechnie (2015) including commentary and an integration of data. Journal of Archaeological Science 58: 184–186 10.1016/j.jas.2015.03.011

McKechnie I, St. Claire D, Salmen-Hartley J. 2019. Kakmakimilh - (306T) - Keith Island 2017/2018 Archaeological Field Program Report. Submitted to Tseshaht First Nation and Pacific Rim National Park Reserve March 7, 2019.

Nichol LM, Doniol-Valcroze T, Watson JC, Rechsteiner Foster EU. 2020. Trends in growth of the sea otter (*Enhydra lutris*) population in British Columbia 1977 to 2017. Canadian Science Advisory Secretariat Pacific Region Science Advisory Report 2020/036. Department of Fisheries and Oceans Canada Pacific Region, Nanaimo.

Rechsteiner EU, Watson JC, Tinker MT, Nichol LM, Henderson MJM, McMillan CJ, DeRoos M, Fournier MC, Salomon AK, Honka LD, Darimont CT. 2019. Sex and occupation time influence niche space of a recovering keystone predator. Ecology and Evolution 9: 3321–3334

Singh GG, McKechnie I. 2015. Making the Most of Fragments: A Method for Estimating Shell Length From Fragmentary Mussels (*Mytilus californianus and M. trossulus*) on the Pacific Coast of North America. Journal of Archaeological Science 58: 175–183 1016/j.jas.2015.02.029

Slade ES. 2019. Estimating Historic Sea Otter Prevalence from Archaeological and Contemporary California Mussel Size Structure. MRM Thesis School of Resource and Environmental Management: Simon Fraser University.
